# Supplementary material for: Sensitivity to cuticular hydrocarbons across the odorant receptor family in the Indian jumping ant, Harpegnathos saltator
Source: Front Insect Sci. 2026 Mar 23;6:1666444. doi: 10.3389/finsc.2026.1666444 (PMC13050904; doi:10.3389/finsc.2026.1666444)
Supplement: Supplementary file 1 [file DataSheet1.pdf]

Target Gene	Forward Primer (5' to 3')	Reverse Primer (5' to 3')
HsOr219	CACCATGAGTCTATCAATTGTTTATATCTTGGGC	CTAAGCGGCTGAGCTTTGAAGCACC
HsOr228	CACCATGGAACTGCTTTCGTTGAATTTTTTCA	TCAGGACTGTTGCAGCACGTTGA
HsOr240	CACCATGCACATACTTTCATTGACTTTTGCTCT	CTAAGAATCTTTCAGAAAGTTGTACGTCGAATA
HsOr191	CACCATGTGTTTCAGCCAAGTTGTCTTC	TTATTTAGACTCGTCCAGTGATTGCAAA
HsOr213	CACCATGGCGAGAAAAGTAACTCCAAAAGT	TTAACTTTTAGTTGCGTCGTCCTC
HsOr42	CACCATGTCGTCGGTCGACCGT	TTACGCCGCTACCATTCGAATGAAA
HsOr16	CACCATGCAACAGAGCATCCAGCTGAA	TTACGAAGTTAATGTGCGTAACATGTTC
HsOr62	CACCATGGACTCGAACGCGCAGTG	TCACGTTTCTAACATCACTCTTAATACCGAC
HsOr72	CACCATGCCGGATGATCGCTG	TTATTCGTTCATCAACGTTACTCGCAAG
HsOr70	CACCATGACAAGTGAACGATGGAACGA	TTATGTTTCTACCATTACTCGAAGTACTGACAA
HsOr187	CACCATGAAAGTGAACCGGGTGG	TCACATAAAGTTGCGCAATATGGATAAGTA
HsOr186	CACCATGGCATATGCGAATTTTTACGAAGTC	TCACAACAGAGTACGCAGCACC
HsOr180	CACCATGTCGACGTTGGGGCTC	TTACAAAAAAGAACGCAGCACCGACA
HsOr115	CACCATGCTAAAAATCATTGGTTTGTGG	TTATGAATTTTGCTTGGCAAGTAACA
HsOr129	CACCATGGACTGTCTGAGCACCTTTG	TCATTGCATCGCCATTAATACCGAGATG
HsOr139	CACCATGGCGTTGAGTACAGCTCAGG	TCAATACATCGCCAAGAGCACCGA
HsOr152	CACCATGTCGTTCATCCTGATCGTG	TCAATACGTCGCATTCAAGACGGAC

Supplementary Table S1. Primer Sequences used to clone full-length HsOrs. All genes above were cloned via the pENTR™/D-TOPO™ Cloning Kit (Invitrogen, ThermoFisher Scientific, Waltham, MA, USA). All forward primers include a 5' CACC to facilitate directional cloning into the entry vector.
