# Supplementary material for: Sensitivity to cuticular hydrocarbons across the odorant receptor family in the Indian jumping ant, Harpegnathos saltator
Source: Front Insect Sci. 2026 Mar 23;6:1666444. doi: 10.3389/finsc.2026.1666444 (PMC13050904; doi:10.3389/finsc.2026.1666444)
Supplement: Supplementary file 2 [file DataSheet2.pdf]

Order in panel	Compound Name	Common Name	Supplier
**1**	Paraffin Oil		Avantor
**2**	2-Heptanone		Alfa Aesar
**3**	Ethyl Acetate		Acros Organics
**4**	Geranyl Acetate		Alfa Aesar
**5**	(*E*)-2-Hexanal		Alfa Aesar
**6**	1-Octen-3-ol		Alfa Aesar
**7**	Pentane	C5	Fisher Scientific
**8**	Decane	C10	Alfa Aesar
**9**	Undecane	C11	Acros Organics
**10**	Dodecane	C12	Alfa Aesar
**11**	Tridecane	C13	Acros Organics
**12**	Tetradecane	C14	Acros Organics
**13**	Pentadecane	C15	Acros Organics
**14**	Hexadecane	C16	Alfa Aesar
**15**	Heptadecane	C17	Acros Organics
**16**	Octadecane	C18	Acros Organics
**17**	Nonadecane	C19	Alfa Aesar
**18**	Eicosane	C20	Alfa Aesar
**19**	Heneicosane	C21	Acros Organics
**20**	Docosane	C22	Acros Organics
**21**	Tricosane	C23	Alfa Aesar
**22**	Tetracosane	C24	Alfa Aesar
**23**	Pentacosane	C25	Sigma-Aldrich
**24**	Hexacosane	C26	Alfa Aesar
**25**	Heptacosane	C27	Alfa Aesar
**26**	Octacosane	C28	BeanTown Chemical
**27**	Nonacosane	C29	Sigma-Aldrich
**28**	Triacontane	C30	Acros Organics
**29**	Hentriacontane	C31	TCI
**30**	Dotriacontane	C32	Acros Organics
**31**	Tritriacontane	C33	TCI
**32**	Tetratriacontane	C34	Acros Organics
**33**	Pentatriacontane	C35	TCI
**34**	Hexatriacontane	C36	Alfa Aesar, TCI
**35**	Heptatriacontane	C37	Sigma-Aldrich
**36**	2-Methyloctacosane	2-MeC28	J.G. Millar
**37**	(Z)-9-Nonacosene	9-C29:1	J.G. Millar
**38**	3-Methylnonacosane	3-MeC29	J.G. Millar
**39**	13-Methylnonacosane	13-MeC29	J.G. Millar
**40**	15-Methylnonacosane	15-MeC29	J.G. Millar
**41**	2-Methyltriacontane	2-MeC30	J.G. Millar
**42**	(Z)-9-Hentriacontene	9-C31:1	J.G. Millar
**43**	5-Methylhentriacontane	5-MeC31	J.G. Millar
**44**	13-Methylhentriacontane	13-MeC31	J.G. Millar
**45**	15-Methylhentriacontane	15-MeC31	J.G. Millar
**46**	13,23-Dimethylheptatriacontane	13,23-DiMeC37	J.G. Millar

Supplementary Table S2. Hydrocarbon panel used for functional characterization of HsOrs. The first 6 odorants are the diagnostic panel used to confirm ab2 sensillum identity. Pentane (7) is the solvent for the other hydrocarbons and it is the vehicle control. The test panel compounds used for analysis of hydrocarbon response are compounds 8-46.
